# Symptoms of anxiety and depression predicting fall-related outcomes among older Americans: a longitudinal study

**DOI:** 10.1186/s12877-022-03406-8

**Published:** 2022-09-14

**Authors:** Yuqian Luo, Christina E. Miyawaki, Maritta A. Valimaki, Siyuan Tang, Hongyu Sun, Minhui Liu

**Affiliations:** 1 Xiangya School of Nursing, Central South University, 172 Tongzipo Road of Yuelu District, Changsha, 410013 China; 2 University of Houston Graduate College of Social Work, Houston, TX USA; 3 Department of Nursing Science, University of Turku, Turku, Finland; 4 School of Nursing, Peking University, Beijing, China

**Keywords:** Fall, Fear of falling, Activity restrictions, Anxiety symptoms, Depressive symptoms, Older adult

## Abstract

**Background:**

Anxiety and depressive symptoms are associated with fear of falling and fear of falling-related activity restrictions. However, it remains unknown whether anxiety or depressive symptoms alone could predict fear of falling and activity restrictions in older adults. We sought to determine if anxiety and depressive symptoms alone could be an independent predictor of fear of falling and activity restrictions in community-dwelling older adults.

**Methods:**

This longitudinal analysis used waves 5 (time 1, [T1]) and 6 (time 2, [T2], 1 year from T1) data (*N* = 6376) from the National Health and Aging Trends Study. The Generalized Anxiety Disorder Scale 2 and Patient Health Questionnaire 2 were used to assess anxiety and depressive symptoms, respectively. Interview questions included demographics, health-related data, and fall worry levels (no fear of falling, fear of falling but no activity restrictions, and activity restrictions). Using multinomial logistic regression models, we examined whether anxiety and depressive symptoms (T1) predicted fear of falling and activity restrictions (T2).

**Results:**

In wave 5 (T1, mean age: 78 years, 58.1% female), 10 and 13% of participants reported anxiety and depressive symptoms. About 19% of participants experienced fear of falling but not activity restrictions, and 10% of participants developed activity restrictions in wave 6 (T2), respectively. Participants with anxiety symptoms at T1 had a 1.33 times higher risk of fear of falling (95% CI = 1.02–1.72) and 1.41 times higher risk of activity restrictions (95% CI = 1.04–1.90) at T2. However, having depressive symptoms did not show any significance after adjusting for anxiety symptoms.

**Conclusions:**

Anxiety symptoms seemed to be an independent risk factor for future fear of falling and activity restrictions, while depressive symptoms were not. To prevent future fear of falling and activity restrictions, we should pay special attention to older individuals with anxiety symptoms.

**Supplementary Information:**

The online version contains supplementary material available at 10.1186/s12877-022-03406-8.

## Background

Fear of falling has become a major public health issue among older adults over the past decades, with 21 to 85% having experienced fear of falling with or without falls [1, 2]. Fear of falling is commonly defined as the feeling of being “afraid or shaky” about falling [3]. Although fear of falling may promote fall prevention behaviors, excessive fear of falling may decrease people’s mobility, increase the possibility of falls or injuries, result in social isolation, and lead to fear of falling-related activity restrictions [4–7]. Activity restrictions can further result in impaired balance, functional impairment, and actual falls [7–9]. Given these negative outcomes, identifying risk factors of fear of falling and activity restrictions is a significant public health concern.

Anxiety and depressive symptoms are the most common psychological symptoms associated with fear of falling and activity restrictions [9–11], and their prevalence among older adults in the United States can be up to 20 and 49%, respectively [12–15]. Most previous studies investigated anxiety and depressive symptoms as an overall condition or focused on one without considering controlling for the other as a confounding variable. These two symptoms shared mild primary symptoms such as fatigue or poor attention in the early stage, and they had a high comorbidity rate of 13 to 30% [16–20]. However, it is essential to differentiate these two concepts and explore their independent effects on future fear of falling and activity restrictions.

From the clinical point of view, anxiety and depressive symptoms were two different concepts. According to the Chinese Classification and Diagnostic Criteria of Mental Disorders, 3rd Edition (CCMD-3), anxiety symptoms were upwardly hyperactive and characterized mainly by nervousness without a clear object or specific content. Depressive symptoms were inhibited downward and dominated by a depressed state of mind that could range from grumpy or grief [21]. A prospective study showed that a state of anxiety symptoms alone may be a precursor to developing either depressive symptoms alone or comorbid symptoms of anxiety and depression [22]. This suggests that anxiety symptoms need to be separated from depressive symptoms. If the anxiety symptoms alone could be detected and treated, it may be possible to prevent depressive symptoms or comorbid symptoms of anxiety and depression, and reduce falls, other health side effects, and even suicide rate [23].

Several previous studies have indicated that anxiety symptoms are a significant predicting factor for fear of falling and activity restrictions without controlling for depressive symptoms [10, 24–26]. Only one small cross-sectional study (*N* = 25) examined the association between anxiety symptoms and fear of falling controlling for depressive symptoms [27]. An extensive body of literature has also identified depressive symptoms as a risk factor for fear of falling and activity restrictions [7, 8, 25, 28], and contributed to the understanding of the bidirectional, mutually reinforcing relationship between depressive symptoms and fear of falling, and/or activity restrictions [29, 30]. But none of the studies controlled for anxiety symptoms.

Therefore, the purpose of this longitudinal study was to examine the independent effects of anxiety and depressive symptoms on fear of falling and activity restrictions among community-dwelling older adults in the United States (U.S.). We hypothesized that both anxiety and depressive symptoms can be independent predictors of fear of falling and activity restrictions in older adults.

## Methods

### Study design, setting, and sample

We used data from the National Health and Aging Trends Study (NHATS) wave 5 (2015) and wave 6 (2016). Since 2011, the NHATS has collected health information from a nationally representative sample of Medicare beneficiaries aged 65 and older in the U.S. [31], and the sample was replenished in 2015. Interviews were conducted annually to document the changes over time. In our study, a total of 6397 participants completed the interviews in both waves (time 1 [T1] and time 2 [T2]). We excluded 21 beneficiaries who had incomplete data on fear of falling or activity restrictions. Thus, a total of 6376 beneficiaries formed the final sample.

### Measurements

#### Independent variables

Anxiety symptoms were measured by the 2-item Generalized Anxiety Disorder Scale (GAD-2) [31]. It screens anxiety symptoms by asking, “Over the last month, how often have you (a) felt nervous, anxious, or on edge, and (b) been unable to stop or control worrying?” Response options were “1=not at all”, “2 = several days”, “3=more than half the days”, and “4=nearly every day.” The total score ranges from two to eight, with the cut-off point of five or higher indicating the presence of anxiety symptoms [32].

Depressive symptoms were measured using the 2-item Patient Health Questionnaire (PHQ-2) [31]. PHQ-2 measures depressive symptoms by asking, “Over the last month, how often have you (a) had little interest or pleasure in doing things, and (b) felt down, depressed, or hopeless?” Responses were based on a four-point scale (1 = not at all, 2 = several days, 3 = more than half the days, 4 = nearly every day). The total score ranges from two to eight, and a total score of five or higher was used to indicate the presence of depressive symptoms [33].

#### Dependent variables

Fear of falling and activity restrictions were assessed by the two questions, “In the last month, did you worry about falling down?” If the answer was yes, then participants were asked, “In the last month, did this worry ever limit your activities?” Based on the participants’ responses, a three-category variable was created to indicate the fall worry levels: No fear of falling (coded 0), had fear of falling but not activity restrictions (coded 1) and had fear of falling-related activity restrictions (coded 2).

### Covariates

We included covariates hypothesized to be associated with our outcomes of interest. These included demographic variables such as age, gender (female vs. male); race/ethnicity (non-Hispanic White, non-Hispanic Black, Hispanic, all other); education level (less than high school, high school graduates, some college or vocational school, bachelor’s degree or higher); and living arrangement (alone, with spouse/partner only, with others only, with spouse/partner and others).

Health related covariables included (a) the number of chronic illnesses (heart attack/heart disease, high blood pressure, arthritis, osteoporosis, diabetes, lung disease, stroke, and cancer); (b) whether the participants had dementia (yes/no); (c) the number of activities of daily living (ADL) impairment, ranging from 0 to 4 (feeding, bathing, toileting, dressing); (d) the number of instrumental activities of daily living (IADL) impairment, ranging from 0 to 7 (bed transfer, moving inside the house, doing laundry, shopping, preparing meals, taking medication, and managing money); (e) whether the participants were bothered by pain in the last month (yes/no); (f) body mass index (BMI) of the participants (normal/obesity [≥30 kg/m2]); (g) whether the participants were hospitalized over the past year (yes/no); and (h) whether the participants had problems with balance or coordination in the last month (yes/no).

### Statistical analyses

Continuous variables were presented as mean ± standard deviations and categorical variables as frequencies and percentages. We used two-sample t-tests to estimate the distribution of age, ADLs, and IADLs across the fall worry levels. Chi-square tests were used to test the differences among groups of fall worry levels for categorical variables.

To determine whether anxiety and/or depressive symptoms at T1 could predict the fall worry levels at T2 independently, we performed three sets of multinominal logistic regression models in three steps. First, we modeled the effects of anxiety symptoms at T1 on fall worry levels at T2, in which fall worry levels at wave 6 were treated as the outcome while fall worry levels at T1 were controlled. Next, we conducted similar models replacing anxiety symptoms with depressive symptoms as the main predictor to examine the effects of depressive symptoms on fall worry levels. Finally, we examined the independent effects of anxiety and depressive symptoms on fall worry levels by including them as main predictors simultaneously in the models. In each model set, we first estimated the crude effects (Model 1), followed by the effects adjusted for demographic variables (Model 2), and finally the effects adjusted for demographic and health-related covariates (Model 3).

To improve the robustness of the results, we performed a sensitivity analysis excluding samples who were interviewed by proxy (*n* = 439). Due to the small proportion of missing data and the large sample size, we did not use any techniques to handle the missing data. For all models, relative risk ratios (RRR) and 95% confidence intervals were reported. *P* values less than 0.05 indicated statistical significance. All analyses were conducted using Stata/SE 15.0 (Stata Corp., College Station, TX).

## Results

Table 1 summarized the baseline characteristics of the participants based on their fall worry levels. The sample (*N* = 6376) was on average 78 ± 7.73 years old. The majority were non-Hispanic White (69.1%) and female (58.1%) with more than half of them (54%) having college and above education. About 10 and 14% of participants reported anxiety and depressive symptoms at T1 respectively. Three hundred one participants experienced both anxiety and depressive sympotms. About 21% (*n* = 1353) of participants had fear of falling and 11% (*n* = 698) had activity restrictions at T2 (Table 1) exclusively. Participants with activity restrictions were the oldest among the three groups. They also had the highest number of ADL and IADL impairment, hospitalization, and falls, and suffered from pain.

**Table 1:** Baseline characteristics of participants stratified by fall worry levels at T1 (*N* = 6202 ~ 6376)

Characteristics	No fear of falling (*n* = 4513, 70.8%)	Had fear of falling but not activity restrictions (*n* = 1215, 19.0%)	Had activity restrictions (*n* = 648, 10.2%)	***P*** values
**Age, M ± SD**	77.1 ± 7.48	79.9 ± 7.83	80.5 ± 8.13	.005
**Sex, n (%)**				<.001
Female	2455 (54.4)	816 (67.2)	434 (67.0)	
Male	2058 (45.6)	399 ((32.8)	214 (33.0)	
**Race/ethnicity, n (%)**				<.001
White, non-Hispanic	3059 (67.8)	893 (73.5)	454 (70.0)	
Black, non-Hispanic	1011 (22.4)	187 (15.4)	105 (16.2)	
Hispanic	232 (5.1)	83 (6.8)	51 (7.9)	
Other	211 (4.7)	52 (4.3)	38 (5.9)	
**Education, n (%)**				.001
Less than high school	890 (20.1)	278 (23.2)	168 (26.7)	
High school graduate	1155 (26.1)	333 (27.8)	162 (25.7)	
Some college or vocational school	1185 (26.8)	300 (25.1)	162 (25.7)	
College or higher	1196 (27.0)	285 (23.8)	138 (21.9)	
**Living arrangement, n (%)**				<.001
Alone	1449 (32.1)	476 (39.2)	258 (39.8)	
With spouse/partner only	1862 (41.3)	417 (34.3)	195 (30.1)	
With others only	741 (16.4)	238 (19.6)	152 (23.5)	
With spouse/partner and others	461 (10.2)	84 (6.9)	43 (6.6)	
**BMI, n (%)**				<.001
Normal (< 30 kg/m^2^)	3145 (71.4)	783 (66.6)	393 (63.3)	
Obese (≥30 kg/m^2^)	1261 (28.6)	392 (33.4)	228 (36.7)	
**Pain, n (%)**				<.001
No	2346 (52.0)	405 (33.4)	144 (22.3)	
Yes	2162 (48.0)	807 (66.6)	503 (77.7)	
**Number of ADL impairment, M ± SD**	0.36 ± 0.86	0.66 ± 1.07	1.53 ± 1.36	<.001
**Number of IADL impairment, M ± SD**	0.68 ± 1.27	1.25 ± 1.55	2.38 ± 1.70	<.001
**Hospitalization, n (%)**				<.001
No	3692 (81.9)	907 (74.9)	421 (65.4)	
Yes	816 (18.1)	304 (25.1)	223 (34.6)	
**Fall history, n (%)**				<.001
No	3353 (74.4)	664 (54.8)	288 (44.4)	
Yes	1154 (25.6)	548 (45.2)	360 (55.6)	
**Dementia, n (%)**				<.001
No	4281 (94.9)	1144 (94.5)	561 (86.7)	
Yes	228 (5.1)	67 (5.5)	86 (13.3)	
**Number of chronic illnesses, n (%)**				<.001
0	438 (9.7)	33 (2.7)	11 (1.7)	
1–3	3205 (71.0)	806 (66.3)	356 (54.9)	
4+	870 (19.3)	376 (31.0)	281 (43.4)	
**Problems with balance, n (%)**				<.001
No	3587 (79.5)	564 (46.4)	123 (19.0)	
Yes	923 (20.5)	651 (53.6)	523 (81.0)	
**Anxiety symptoms, n (%)**				<.001
No	4200 (93.6)	1019 (84.8)	469 (73.2)	
Yes	286 (6.4)	183 (15.2)	172 (26.8)	
**Depressive symptoms, n (%)**				<.001
No	4085 (91.2)	1016 (84.7)	420 (65.6)	
Yes	392 (8.8)	184 (15.3)	220 (34.4)	

*Abbreviations*: *T1* time 1, *M* mean, *SD* standard deviations, *BMI* body mass index, *ADL* activities of daily living, *IADL* instrument activities of daily living

**Fig. 1:**
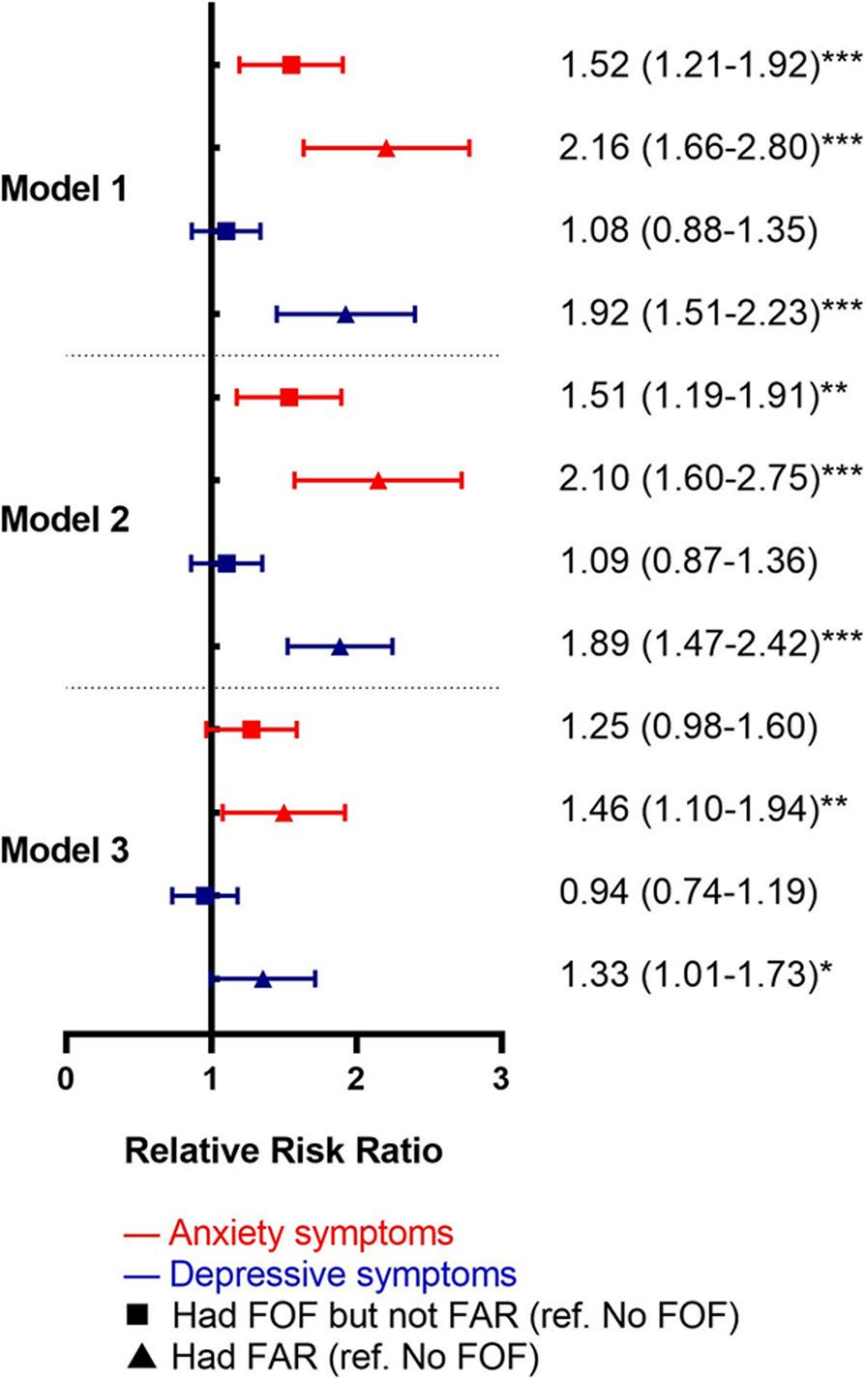
Results of anxiety and depressive symptoms on fall worry levels without controlling for each other. Legends: * *P* < .05, ** *P* < .01, ***P < .001. Abbreviation: FOF = fear of falling; FAR = fear of falling related activity restrictions. Model 1 adjusted for fall worry level at T1; Model 2: Model 1 + demographic covariables (age, gender, race/ethnicity, education, living arrangement); Model 3: Model 2 + health-related covariables (BMI, pain, ADL, IADL, hospitalization, falls, balance, number of chronic illnesses)

Figure 1 showed the multinominal logistic regression results of anxiety and depressive symptoms at T1 on fall worry levels at T2 without controlling for each other. The RRR of fall worry levels showed a downtrend from the crude model (Model 1) to the fully adjusted model (Model 3), and a rising trend from the level of “Had fear of falling but not activity restrictions” to “Had activity restrictions”.

For anxiety symptoms, compared to participants without anxiety symptoms, those with anxiety symptoms were more likely to develop fear of falling in the following year in Models 1 and 2 (RRR = 1.52, 95% CI = 1.21–1.92; RRR = 1.51, 95% CI = 1.19–1.91, respectively), but results became insignificant in the fully adjusted model (Model 3, RRR = 1.25, 95% CI = 0.98–1.60). The RRR of “Had activity restrictions” was 1.46 times greater among participants with anxiety symptoms compared to those without anxiety symptoms (95% CI = 1.10–1.94). For participants with depressive symptoms, the results of “Had fear of falling but not activity restrictions” showed insignificant in the crude model (Model 1). However, the RRR of “Had activity restrictions” became 1.33 times greater compared to those without depressive symptoms in the fully adjusted model (Model 3, 95% CI = 1.01–1.73).

**Fig. 2:**
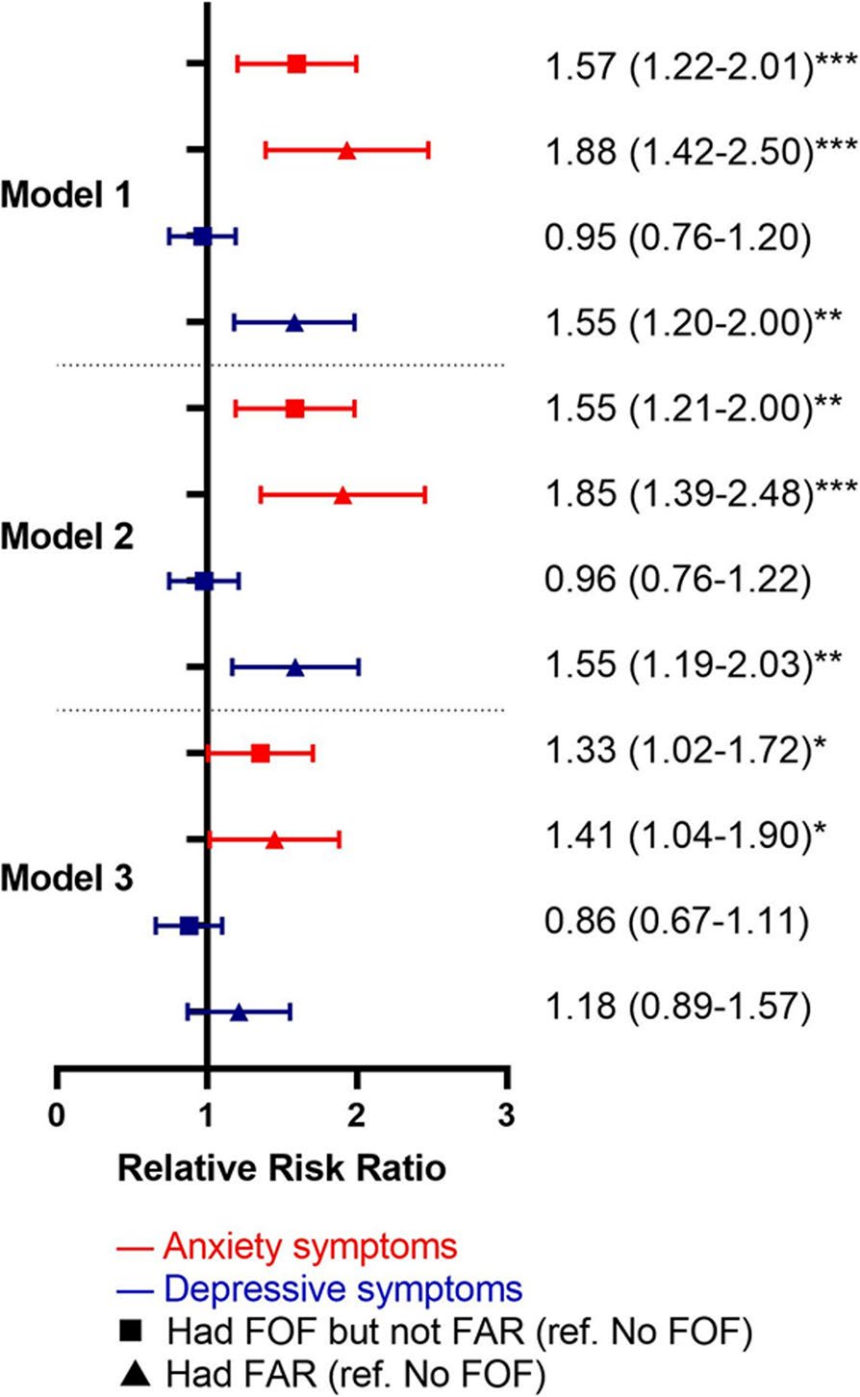
Results of anxiety and depressive symptoms on fall worry levels controlling for each other. Legends: * *P* < .05, ** *P* < .01, ***P < .001. Abbreviation: FOF = fear of falling; FAR = fear of falling related activity restrictions. Model 1 adjusted for fall worry levels and depressive symptoms/anxiety symptoms at T1; Model 2: Model 1 + demographic covariables (age, gender, race/ethnicity, education, living arrangement); Model 3: Model 2 + health-related covariables (BMI, pain, ADL, IADL, hospitalization, falls, balance, number of chronic illnesses)

Figure 2 indicated the associations of anxiety and depressive symptoms at T1 on fall worry levels at T2 controlling for each other. For anxiety symptoms, the results were almost all statistically significant even after controlling for depressive symptoms. Participants with anxiety symptoms had 1.33 and 1.41 times increased risk of “Had fear of falling but not activity restrictions” and “Had activity restrictions” 1 year later compared to those without anxiety symptoms (Model 3, 95% CI = 1.02–1.72, 1.04–1.90, respectively). However, depressive symptoms showed different patterns. Depressive symptoms presented no statistically significant results in all models at “Had fear of falling but not activity restrictions” in the following year after controlling for anxiety symptoms. The results only showed participants with depressive symptoms had 1.55 times increased risk of having activity restrictions in Models 1 and 2 (95% CI = 1.20–2.00, 1.19–2.03, respectively), but became insignificant in Model 3 (RRR = 1.18; 95% CI = 0.89–1.57). Depressive symptoms were less likely to have an independent predicting impact on fear of falling and activity restrictions compared to anxiety symptoms.

The results of sensitivity analyses limiting the participants to non-proxy participants only showed similar results to our earlier analyses, supporting that anxiety symptoms were an independent factor of future fear of falling and activity restrictions. (see Supplemental Table 1).

## Discussion

In this large cohort study, we investigated the independent effects of anxiety and depressive symptoms on fear of falling and activity restrictions using a nationally representative sample of older adults in the United States. We found that (1) 10 and 13% of participants at T1 reported experiencing symptoms of anxiety or depression, and approximately 20 and 11% reported “Had fear of falling but not activity restrictions” or “Had activity restrictions” at T2, respectively; (2) anxiety symptoms were associated with a higher risk ratio of “Had fear of falling but not activity restrictions” and “Had activity restrictions” 1 year later adjusting for depressive symptoms; and (3) depressive symptoms did not seem to have any association with fear of falling and activity restrictions.

Consistent with previous studies [12, 34], our results showed that anxiety symptoms increased the risk of future fear of falling. The reason could be that since fear has an object whereas anxiety can happen without an object. The presence of anxiety symptoms can be the driving factor for fear of falling [34]. Moreover, we found that anxiety symptoms remained a significant independent factor that is associated with “Had fear of falling but not activity restrictions” even after controlling for depressive symptoms. More specifically, participants with anxiety symptoms were 1.33 times more likely to develop fear of falling in the following year compared to those without anxiety symptoms. This result was consistent with Payette et al’s study [27], a pilot study that concluded the significant relationship between anxiety symptoms and fear of falling while controlling for depressive symptoms, fall risk, and sociodemographic variables. Payette’s study was a small, pilot study (*N* = 25) with a high proportion of women (88%). However, we can say that the results of our study can be better interpreted and generalizable because of the large sample size and a more balanced female proportion (58.1%) [27].

We also found that anxiety symptoms increased the risk of future activity restrictions independently. This result also supported the previous studies [25, 26]. For example, Hull and colleagues found that anxiety symptoms measured by the Geriatric Anxiety Inventory were a significant factor of fall-related outcome expectancy in a sample of 205 community-dwelling older adults, and activity restrictions was a component of this outcome index [25]. Painter and colleagues found anxiety symptoms assessed by the Hamilton Anxiety Scale could predict activity restrictions through the indirect influence of fear of falling rather than directly among 99 community-dwelling older adults [26]. Though these studies found a significant association between anxiety symptoms and activity restrictions, it should be noted that they failed to establish a direct, independent, and longitudinal association. First, they did not use activity restrictions as a direct target outcome but measured it via other variables such as fear of falling. Second, they did not control for depressive symptoms. Third, the longitudinal association could not be established due to their cross-sectional study design. Our study not only provided evidence of whether anxiety symptoms were directly and independently related to activity restrictions, but presented the significant longitudinal effects of anxiety symptoms on future fall worry levels. Therefore, our results underscored the importance of screening older adults with anxiety symptoms to prevent future development of fear of falling and activity restrictions, as well as related adverse health outcomes.

Our results showed that depressive symptoms had no significant associations with fear of falling and activity restrictions in the fully adjusted model. This result was a surprise because previous studies showed opposite results [24]. For example, Namkee and colleagues found “onset of depression” (no depression at baseline but depression 1 year later) and “continued depression” (had depression at baseline and continued at 1 year later) were significantly associated with greater odds of activity restrictions at 1 year after the baseline [30]. We speculated that the significant correlation in this study was because both “onset of depression” and “continued depression” were more focused on the concurrent relationship between depressive symptoms and activity restrictions, as opposed to our focus on the longitudinal association between depressive symptoms at baseline and activity restrictions 1 year later. Please be noted that depressive symptoms in our study were measured via PHQ-2 which may have lower acceptable accuracy for screening for depression compared to other tools (36) (e.g., PHQ-9) commonly used in studies investigating the association between depressive symptoms and fall-related outcomes. This could also explain the opposite results.

The different results of the association between depressive symptoms and activity restrictions with or without controlling for anxiety symptoms should be highlighted. Taking anxiety symptoms into account, the association between depressive symptoms and activity restrictions became insignificant, suggesting anxiety symptoms may weaken the effects of depressive symptoms on activity restrictions. Further investigations are needed to clarify the role of anxiety symptoms on the pathway from depressive symptoms to activity restrictions and determine its effect size.

Our findings provided some evidence that anxiety symptoms may be able to predict future levels of fear of falling and activity restrictions. This finding has implications for guiding the strategies for preventing fear of falling and activity restrictions among older adults from the context of tertiary prevention. First, the knowledge of the scientific relationship between anxiety symptoms and future levels of fear of falling and activity restrictions should be disseminated especially to older adults and their caregivers. They should be aware that anxiety symptoms affect future fear of falling and activity restrictions levels, and subsequently may lead to more adverse health outcomes such as falls and functional impairment. Second, anxiety symptoms should be screened for early detection and diagnosis. Then we can identify and treat older adults with a high risk of developing fear of falling and activity restrictions better as secondary prevention. The assessment tool used in this study, GAD-2 is readily available and easy to use in the community [12] and therefore, may benefit the public. Finally, older adults who already have been evaluated for anxiety symptoms should receive timely and effective targeted interventions. The issue of the high comorbidity of anxiety and depressive symptoms should not be ignored. Using multiple years of follow-up measurements, as well as different measurement tools, further investigations are needed to examine the effect size of anxiety and depressive symptoms.

The strengths of this study include its longitudinal design and a large nationally representative sample of the study population. However, several study limitations should be noted. Selected variables were all based on retrospective self-reported interviews. Recall bias may influence the accuracy of the data and may have caused a lack of significant associations between depressive symptoms with fear of falling and activity restrictions. The GAD-2 and PHQ-2 were used to measure participants’ anxiety and depressive symptoms respectively. These tools were originally developed for not diagnostic but screening purposes. Therefore, the data may be limited to the state of emotion at the time of the interview.

## Conclusions

Based on the nationally representative sample of Medicare beneficiaries, we found that anxiety symptoms were an independent risk factor for potential future fear of falling and activity restrictions. Depressive symptoms did not have any associations with fear of falling and activity restrictions. As a future strategy for preventing fear of falling and activity restrictions, special attention should be paid to older adults with anxiety symptoms in the communities.

## Supplementary Information

**Additional file 1: Supplemental Table S1.** Independent effects of anxiety and depressive symptoms at T1 and on fall worry levels at T2 controlling for each other, excluding proxy respondents. (*N* = 5, 937).

## Data Availability

The data sets analyzed in the current study are publicly available: NHATS (https://www.nhats.org/).

## Abbreviations

T1: Time
T2: Time 2, one year after time 1
NHATS: National Health and Aging Trends Study
U.S.: United States
GAD-2: Generalized Anxiety Disorder Scale-2
PHQ-2: Patient Health Questionnaire-2
ADL: Activities of Daily Living
IADL: Instrumental Activities of Daily Living
RRR: Relative Risk Ratio
CI: Confidence Interval
