# Supplementary material for: Symptoms of anxiety and depression predicting fall-related outcomes among older Americans: a longitudinal study
Source: BMC Geriatr. 2022 Sep 14;22:749. doi: 10.1186/s12877-022-03406-8 (PMC9472405; doi:10.1186/s12877-022-03406-8)
Supplement: Supplementary file 1 — Additional file 1: Supplemental Table S1. Independent effects of anxiety and depressive symptoms at T1 and on fall worry levels at T2 controlling for each other, excluding proxy respondents. (N = 5, 937). [file 12877_2022_3406_MOESM1_ESM.docx]

***Supplemental Table S1.* Independent effects of anxiety and depressive symptoms at T1 and on fall worry levels at T2 controlling for each other, excluding proxy respondents.** **(N = 5, 937)**

| **Variables** | **Model 1**  **RRR (95% CI)** | **Model 2**  **RRR (95% CI)** | **Model 3**  **RRR (95% CI)** |
| --- | --- | --- | --- |
| **Anxiety symptoms** |  | | |
| Had fear of falling but not not activity restrictions  (*ref*. No fear of falling) | 1.67 (1.28-2.17) *** | 1.70 (1.31-2.22) *** | 1.45 (1.10-1.90) ** |
| Had activity restrictions  (*ref*. No fear of falling) | 1.87 (1.37-2.55) *** | 1.94 (1.41-2.66) *** | 1.51 (1.09-2.09) * |
| **Depressive symptoms** |  |  |  |
| Had fear of falling but not not activity restrictions  (*ref*. No fear of falling) | 1.01 (0.79-1.29) | 1.05 (0.82-1.35) | 0.90 (0.69-1.18) |
| Had activity restrictions  (*ref*. No fear of falling) | 1.59 (1.20-2.10) ** | 1.60 (1.19-2.14) ** | 1.23 (0.91-1.67) |

* *P* <.05, ** *P* <.01, ****P*<.001.

Model 1: independent variable of interest;

Model 2: Model 1 + demographic covariables (age, gender, race/ethnicity, education, living arrangement);

Model 3: Model 2 + health-related covariables (BMI, pain, ADL, IADL, hospitalization, falls, balance, number of chronic illnesses).
